# Evidence of Selection Against Damaged Mitochondria During Early Embryogenesis in the Mouse

**DOI:** 10.3389/fgene.2020.00762

**Published:** 2020-07-15

**Authors:** Thiago S. Machado, Carolina H. Macabelli, Maite Del Collado, Flávio V. Meirelles, Francisco E. G. Guimarães, Marcos R. Chiaratti

**Affiliations:** ^1^Departamento de Genética e Evolução, Universidade Federal de São Carlos, São Carlos, Brazil; ^2^Faculdade de Medicina Veterinária e Zootecnia, Universidade de São Paulo, São Paulo, Brazil; ^3^Faculdade de Zootecnia e Engenharia de Alimentos, Universidade de São Paulo, Pirassununga, Brazil; ^4^Instituto de Física de São Carlos, Universidade de São Paulo, São Carlos, Brazil

**Keywords:** mitochondria, embryo, mitochondrial DNA, mtDNA, mouse, cytoplasmic transfer, NZB, photosensitization

## Abstract

There is evidence of a purifying filter acting in the female germline to prevent the expansion of deleterious mutations in the mitochondrial DNA (mtDNA). Given our poor understanding of this filter, here we investigate the competence of the mouse embryo to eliminate dysfunctional mitochondria. Toward that, mitochondria were damaged by photoirradiation of NZB/BINJ zygotes loaded with chloromethyl-X-rosamine (CMXRos). The resultant cytoplasm was then injected into C57BL/6J zygotes to track the levels of NZB/BINJ mtDNA during the preimplantation development. About 30% of NZB/BINJ mtDNA was present after injection, regardless of using photoirradiated or non-photoirradiated cytoplasmic donors. Moreover, injection of photoirradiated-derived cytoplasm did not impact development into blastocysts. However, lower levels of NZB/BINJ mtDNA were present in blastocysts when comparing injection of photoirradiated (24.7% ± 1.43) versus non-photoirradiated (31.4% ± 1.43) cytoplasm. Given that total mtDNA content remained stable between stages (zygotes vs. blastocysts) and treatments (photoirradiated vs. non-photoirradiated), these results indicate that the photoirradiated-derived mtDNA was replaced by recipient mtDNA in blastocysts. Unexpectedly, treatment with rapamycin prevented the drop in NZB/BINJ mtDNA levels associated with injection of photoirradiated cytoplasm. Additionally, analysis of mitochondria-autophagosome colocalization provided no evidence that photoirradiated mitochondria were eliminated by autophagy. In conclusion, our findings give evidence that the mouse embryo is competent to mitigate the levels of damaged mitochondria, which might have implications to the transmission of mtDNA-encoded disease.

## Introduction

Mitochondria play a central role in cellular energy production (i.e., ATP), besides being involved in several other functions including Ca^2+^ buffering, innate immunity, biogenesis of iron-sulfur clusters and apoptosis (Wallace and Chalkia, 2013). Most proteins needed for mitochondrial function are encoded in the nucleus and imported by the organelle (Wallace and Chalkia, 2013). Yet, the mitochondrion also relies on 37 genes (13 mRNAs, 22 tRNAs, and two rRNAs) encoded by its own genome, the mitochondrial DNA (mtDNA). The importance of these genes is revealed by mutations in mtDNA, which can result in mitochondrial dysfunction and severe pathologies in humans (Stewart and Chinnery, 2015). Manifestation of these pathologies is difficult to predict though as it depends on the level of mutant mtDNA. Multiple copies of mtDNA are present in each cell and mutations commonly coexist with wild-type molecules, a condition termed heteroplasmy (Schon et al., 2012; Burr et al., 2018). Given that most mtDNA mutations are recessive, wild-type molecules can complement the mutation defect. A threshold level of mutant mtDNA is needed to impair mitochondrial function (i.e., 60–80%), but this threshold level varies for different mutations and tissues (Schon et al., 2012; Burr et al., 2018).

Due to the lack of efficient methods to treat mitochondrial disease, much attention has been given to prevent its transmission to the next generation. Yet, the non-Mendelian pattern of mtDNA inheritance makes difficult to predict transmission of such disease (Stewart and Chinnery, 2015). Despite few exceptions (Luo et al., 2018), autophagic elimination of paternal mitochondria shortly after fertilization assures mtDNA to be exclusively inherited from the mother (Rojansky et al., 2016; Wei et al., 2020). In addition, in case of heteroplasmy, several mechanisms take place during germline development toward reestablishing homoplasmy (i.e., existence of a single mtDNA genotype, regardless of mutant or wild-type). For instance, the mitochondrial genetic bottleneck allows for quick changes in mtDNA genotype frequency (Jenuth et al., 1996; Cree et al., 2008; Wai et al., 2008; Floros et al., 2018; Latorre-Pellicer et al., 2019). Also, there is increasing evidence in support of purifying selection acting in the female germline to prevent deleterious mutations (i.e., non-synonymous) in mtDNA from accumulating in the population (Burr et al., 2018).

One of the most consistent evidence of purifying selection first came from the work by Stewart et al. (2008). Using a mouse model with a burden of randomly generated mtDNA mutations, the authors found that synonymous mutations in protein-coding genes are preferentially transmitted to offspring than non-synonymous mutations. In addition, mutations in tRNA and rRNA genes were more often present in offspring than mutations in protein-coding genes (Stewart et al., 2008). Similar findings have been reported in mice and humans (Sato et al., 2007; Fan et al., 2008; Freyer et al., 2012; Sharpley et al., 2012; Li et al., 2016; Floros et al., 2018; Latorre-Pellicer et al., 2019; Wei et al., 2019). However, the mechanism underpinning purifying selection is currently unclear (Burr et al., 2018). Here we provide evidence that early embryos mitigate the levels of photoirradiated mitochondria introduced by cytoplasmic transfer (CT), which suggests they are virtually competent to tackle dysfunctional mitochondria harboring deleterious mtDNA mutations.

## Materials and Methods

All chemical and reagents were purchased from Sigma–Aldrich Chemical Co. (St. Louis, MO, United States), unless otherwise stated. All experiments were performed in compliance with the regulations and policies of the National Council for Control of Animal Experimentation (CONCEA, Brazil) and were approved by the Animal Care and Use Committee at Universidade de São Paulo (USP—protocol number 13.1.1832.74.8).

### Source of Mouse and Embryos

Mice containing mtDNA of NZB/BINJ (NZB) origin were obtained by backcrossing NZB females to C57BL/6J (B6) females for five generations. Thereafter, females with NZB mtDNA in a ∼100% B6 background were maintained by brother–sister mating (Machado et al., 2015). Mice containing mtDNA of B6 origin were obtained from F1 females from a cross of B6 females with males of CBA origin. Mice with mtDNA of NZB or B6 origin are hereafter termed NZB and B6, respectively.

To obtain pronuclear zygotes, females were intraperitoneally injected with 5 I.U. of equine chorionic gonadotropin (eCG; Folligon, MSD Animal Health, Summit, United States) and 5 I.U. of human chorionic gonadotropin (hCG; Chorulon, MSD Animal Health), given 46–47 h apart. Immediately after the hCG injection, females were paired with B6 males and inspected for the presence of vaginal plug in the next morning. Pronuclear zygotes were collected from the oviduct (ampulla) of plugged females ∼18 h after the hCG injection using HEPES-buffered KSOM medium (Erbach et al., 1994; Nagy et al., 2003; Machado et al., 2015). Viable zygotes were denuded of cumulus cells by vigorous pipetting in the presence of 0.3% hyaluronidase in HEPES-buffered KSOM. Groups of 20 zygotes were cultured *in vitro* under mineral oil in a 40 μl drop of KSOM. After 96 h of culture in an incubator (set at 37°C, maximum humidity, and 5% CO_2_ in air), embryos were assessed as for the blastocyst rate (Nagy et al., 2003; Machado et al., 2015).

### Induction of Mitochondrial Damage

Chloromethyl-X-rosamine (CMXRos; MitoTracker Red; ThermoFisher Scientific, Waltham, MA, United States) is a mitochondrion-selective fluorescent probe with a strong photosensitizing action (Minamikawa et al., 1999; Lum et al., 2002; Palermo et al., 2002; Lum and Nagley, 2003; Takeuchi et al., 2005). Under photoirradiation, CMXRos absorbs light, leading to excitation of the outer shell electrons and generation of reactive species such as hydroxyl radicals and singlet oxygen within the mitochondrion. These reactive species may damage mitochondrial structures, with evidence of organelle swelling and membrane depolarization (Minamikawa et al., 1999; Lum et al., 2002; Palermo et al., 2002; Lum and Nagley, 2003; Takeuchi et al., 2005).

To induce mitochondrial damage, NZB zygotes at the pronuclear stage were incubated for 30 min with 500 nM CMXRos in HEPES-buffered KSOM at 37°C. Next, based on a previous report (Takeuchi et al., 2005), zygotes were rinsed three times in HEPES-buffered KSOM and photoirradiated for either 0, 2.5, 5, 10, 20, or 60 s. Photoirradiation was performed in groups of 20 zygotes using an inverted microscope (Eclipse TS 100, Nikon Instruments Inc., Tokyo, Japan) equipped with an epifluorescence attachment (50-W mercury burner) with a Texas Red filter (excitation wavelength, 540–580 nm; emission wavelength, 600–660 nm) at 200x magnification (Minamikawa et al., 1999; Lum et al., 2002; Palermo et al., 2002; Takeuchi et al., 2005). Control zygotes were photoirradiated for either 0 or 60 s without prior loading with CMXRos.

### Cytoplasmic Transfer

Five experimental groups were considered during CT experiments: control B6 embryos not subjected to either CMXRos loading or photoirradiation—termed “B6-control”; NZB embryos subjected to CMXRos loading and photoirradiation (P) for either 0 or 20 s—termed “NZB-P0” and “NZB-P20,” respectively; and, B6 embryos subjected to CT using cytoplasm from either NZB-P0 or NZB-P20—termed “CT-P0” and “CT-P20,” respectively.

Micromanipulation was performed using an inverted microscope (Leica DMI RB, Leica, Wetzlar, Germany) equipped with micromanipulators and microinjectors (Narishige, Tokyo, Japan), as previously reported (Machado et al., 2015). Briefly, pronuclear zygotes were incubated for 15 min in HEPES-buffered KSOM medium containing 5 μg/ml cytochalasin and 5 μg/ml nocodazole. Next, ∼30% of cytoplasm was removed from B6 zygotes (calculated as previously reported; Chiaratti et al., 2010), followed by injection in the perivitelline space of a similar amount of cytoplasm derived from NZB zygotes. Pronuclei were always visualized during the micromanipulation procedure to prevent their unintended removal. After micromanipulation, zygotes were placed in an electrofusion solution (0.28 M mannitol, 0.1 mM MgSO_4_, 0.5 mM HEPES, and 0.05% BSA) and subjected to a single electrical pulse of 1 kV/cm (DC) for 45 μs (Multiporator, Eppendorf, Hamburg, Germany) to induce fusion of the NZB cytoplast with the B6 recipient zygote. Successfully fused zygotes were cultured *in vitro* as described above. When applicable, embryos were cultured in the presence of 250 nM rapamycin (Lee et al., 2011; Gilkerson et al., 2012). After 96 h of *in vitro* culture, the blastocyst rate was assessed.

### Evaluation of NZB Levels and Mitochondrial DNA Copy Number

Embryos used for molecular evaluation were sampled immediately before (at the pronuclear stage) or after (at the blastocyst stage) *in vitro* culture. These were rinsed three times in phosphate buffer solution (PBS) containing 0.1% polyvinyl pyrrolidone (PVP) and stored individually in 1 μl PBS plus 0.1% PVP in 0.2 ml tubes at −20°C. Embryos were lyzed for 3 h at 55°C in 50 mM KCl, 10 mM Trix-Cl (pH 8.3), 2 mM MgCl_2_, 0.1 mg/ml gelatin, 0.45% Igepal CA-630, 0.45% Tween 20, and 125 μg/ml proteinase K (ThermoFisher Scientific). Following, lysates were incubated at 95°C for 10 min for proteinase K inactivation, diluted with 45 μl ultrapure H_2_O, and centrifuged at 10,000 × *g* for 5 min. The supernatant was finally used for analysis of NZB levels and mtDNA copy number (Machado et al., 2015).

The levels of NZB mtDNA in zygotes and blastocysts were assessed by quantitative PCR (qPCR) as previously reported by Machado et al. (2015). Briefly, two set of primers were used to amplify either a 118-bp fragment of NZB mtDNA or a 146-bp fragment of B6 mtDNA. Reactions consisted of a final volume of 15 μl containing 5 μl of sample lysate, 200 nM of each primer, and 1x Power SYBR Green Master Mix (ThermoFisher Scientific). Amplifications were performed using the 7500 Fast Real-Time PCR System (ThermoFisher Scientific) and the following cycling conditions: 95°C for 10 min, followed by 40 cycles of 95°C for 15 s, and 62°C for 1 min. SYBR Green fluorescence was read at the end of each extension step. The percentage of NZB mtDNA was calculated in relation to the sum of NZB and B6 mtDNA, as reported by Machado et al. (2015).

Total mtDNA copy number (sum of NZB and B6 mtDNA) in zygotes and blastocysts was assessed as reported by Machado et al. (2015). Toward that aim, a 736-bp fragment of B6 mtDNA was cloned into a plasmid vector (pCR2.1-TopTA; ThermoFisher Scientific). Part of this construct (at concentration of 10^7^, 10^6^, 10^5^, 10^4^, and 10^3^ copies/reaction) was amplified by qPCR in parallel with zygote and blastocyst samples. Conditions of qPCR were the same described above, except for the use of non-discriminative primers that amplify a common fragment (148 bp) from both NZB and B6 mtDNA. The number of mtDNA copies was calculated as reported by Machado et al. (2015).

### Analysis of Mitochondria-Autophagosome Colocalization

Embryos at the two-cell stage (21 h of culture) were fixed in 3.7% paraformaldehyde in PBS with 0.5% Triton X-100 and 0.1% PVP for 15 min at room temperature. Next, embryos were rinsed three times in PBS with 0.1% PVP, and incubated for 1 h at room temperature with a primary antibody (anti-MAP1LC3B raised in rabbit; Cat# L7543, Sigma–Aldrich). Afterward, embryos were rinsed in PBS with 0.1% PVP, and incubated for 1 h at room temperature with an Alexa Fluor 488-tagged secondary antibody raised against rabbit (Cat# A11008, ThermoFisher Scientific). Both antibodies were diluted 1:200 in PBS with 0.1% PVP. Finally, embryos were thoroughly washed in PBS with 0.1% PVP, and mounted on slides with coverslips using Prolong Gold (ThermoFisher Scientific). Embryos were evaluated by confocal microscopy (LSM 780, Zeiss, Oberkochen, Germany) at 1000x magnification. Autophagosomes were visualized at 495 and 519 nm, respectively, for excitation and emission. NZB mitochondria (previously stained with CMXRos for the photosensitization treatment) were visualized at 543 and 580–650 nm, respectively. Images were analyzed using the ZEN lite (Zeiss).

### Statistical Analyses

Statistical analyses were performed using SAS v.9.3 (SAS/STAT, SAS Institute Inc., Cary, NC, United States). When necessary, data were transformed to fit a normal distribution. Data were analyzed by one-way or two-way ANOVA followed by Tukey’s *post hoc* test. Values are reported as mean ± standard error of the mean (SEM).

## Results

### Photoirradiation of CMXRos-Loaded Zygotes Prevents Development Into Blastocysts

**FIGURE 1:**
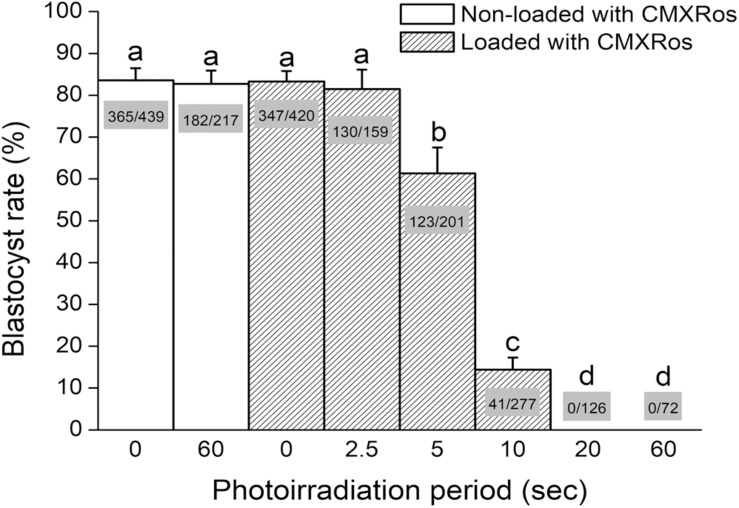
Photoirradiation of CMXRos-loaded zygotes prevents development into blastocysts. Percentage of NZB zygotes that developed into blastocysts after 96 h of *in vitro* culture. Zygotes were either loaded or not with CMXRos prior to photoirradiation. Bar insets represent the number of blastocysts in relation to total number of cultured zygotes. Different letters over bars depict statistical difference (*P* < 0.05).

Aiming to set up photosensitization conditions, zygotes were loaded with CMXRos and photoirradiated for either 0, 2.5, 5, 10, 20, or 60 s before *in vitro* culture and analysis of blastocyst development. Zygotes photoirradiated for either 0 or 60 s, without prior incubation with CMXRos, were used as controls. As a result, photoirradiation of non-loaded zygotes for 60 s did not impact blastocyst rate in comparison with zygotes that were neither incubated with CMXRos nor photoirradiated (Figure 1). Likewise, no effect was seen when CMXRos-loaded zygotes were photoirradiated for 0 or 2.5 s (Figure 1). Yet, photoirradiation for 5 s or more progressively impacted on blastocyst rate (*P* < 0.05); a photoirradiation period of 20 or 60 s was sufficient to completely prevent blastocyst formation (Figure 1). In summary, these findings show a linear impact of photoirradiation time on blastocyst rate, which relied on the prior loading with CMXRos.

### Photoirradiated Mitochondria Injected Into Zygotes Are Selected Against During Early Embryogenesis

**FIGURE 2:**
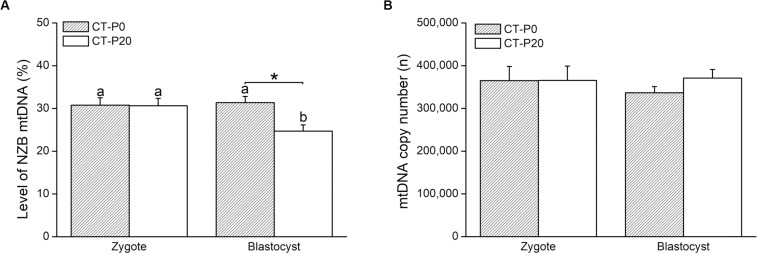
Photoirradiated mitochondria injected into zygotes are selected against during early embryogenesis. CMXRos-loaded zygotes containing mtDNA of NZB origin were photoirradiated for either 0 (P0) or 20 (P20) s to induce mitochondrial damage. Mitochondria from these zygotes were injected by cytoplasmic transfer (CT) into B6 zygotes, resulting in CT-P0 and CT-P20 groups, respectively. CT-derived embryos were assessed at zygote and blastocyst stage as for the levels of NZB **(A)** and total **(B)** mtDNA. Different letters over bars depict statistical difference within group (*P* < 0.05). *Statistical difference within stage (*P* = 0.008).

To investigate whether damaged mitochondria are selectively eliminated during early embryogenesis, donor zygotes (containing NZB mtDNA) were loaded with CMXRos and photoirradiated for either 0 or 20 s. We chose a 20-s exposure time given this was the shortest period that completely precluded development into blastocysts (Figure 1). To validate our system, we first assessed in recipient zygotes (CT-P0 and CT-P20) the levels of NZB mtDNA following CT. As a result, comparable levels (*P* > 0.05) of NZB mtDNA were present in CT-P0 (30.8 ± 1.73) and CT-P20 (30.6 ± 1.73) zygotes (Figure 2A). Similarly, mtDNA copy number (sum of B6 and NZB mtDNA) was not different (*P* > 0.05) between CT-P0 (365,022 ± 33,062) and CT-P20 (365,704 ± 33,314) zygotes (Figure 2B). These zygotes also presented similar mtDNA copy number (*P* > 0.05) compared with zygotes not subjected to CT: B6-control (348,850 ± 23,696), NZB-P0 (375,461 ± 33,388), and NZB-P20 (359,852 ± 23,132). In summary, neither photoirradiation nor CT altered the levels of NZB and total mtDNA in pronuclear zygotes.

We next sought to assess the levels of NZB mtDNA after development of CT-derived zygotes into blastocysts. Toward this, CT-P0 and CT-P20 embryos were cultured *in vitro* for 96 h, reaching the blastocyst stage with similar rates (CT-P0 = 92.3% vs. CT-P20 = 83.8%) when compared to that of B6-control (84.5%) and NZB-P0 (77.9%) embryos. In comparison, only 5.8% of NZB-P20 zygotes developed into blastocysts (*P* < 0.05). In regard of the levels of CT-derived mitochondria, similar (*P* > 0.05) levels of NZB mtDNA were found between blastocysts (31.4% ± 1.43) and zygotes (30.8% ± 1.73) of the CT-P0 group (Figure 2A). Conversely, the levels of NZB mtDNA in CT-P20 embryos dropped (*P* = 0.008) from 30.6% ± 1.73 in zygotes to 24.7% ± 1.43 in blastocysts (Figure 2A). The levels of NZB mtDNA also proved to be lower (*P* < 0.05) in CT-P20 than CT-P0 blastocysts (Figure 2A). On the other hand, mtDNA copy number remained stable (Figure 2B) between groups at the blastocyst stage (CT-P0 = 336,497 ± 14,551 vs. CT-P20 = 371,063 ± 20,054). This was also true when compared with NZB-P0 (352,179 ± 15,704) and B6-control (366,065 ± 11,322) blastocysts. Therefore, these findings provide evidence that photoirradiated mitochondria introduced into zygotes were eliminated during development into blastocysts.

### Rapamycin Treatment Precludes Elimination of Photoirradiated Mitochondria During Early Embryogenesis

**FIGURE 3:**
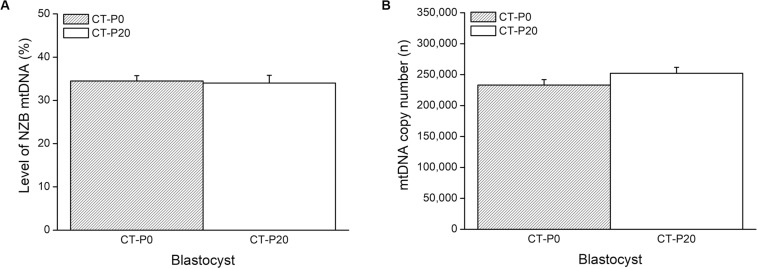
Rapamycin treatment precludes elimination of photoirradiated mitochondria during early embryogenesis. CMXRos-loaded zygotes containing mtDNA of NZB origin were photoirradiated for either 0 (P0) or 20 (P20) s to induce mitochondrial damage. Mitochondria from these zygotes were injected by cytoplasmic transfer (CT) into B6 zygotes, resulting in CT-P0 and CT-P20 groups, respectively. CT-derived blastocysts were assessed as for the levels of NZB **(A)** and total **(B)** mtDNA. No statistical difference (*P* > 0.05).

**FIGURE 4:**
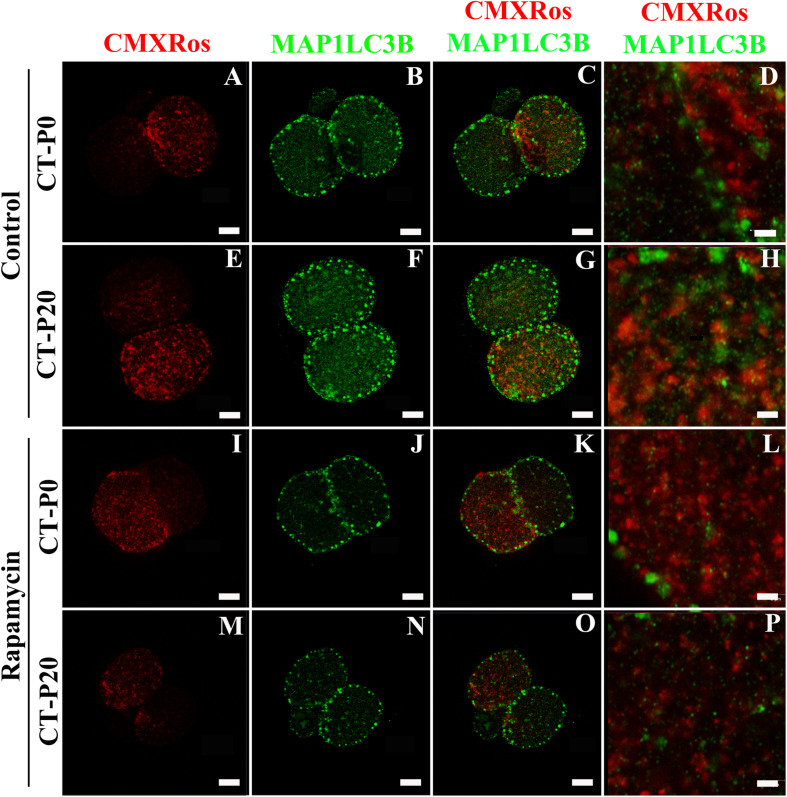
Autophagy is not linked with elimination of photoirradiated mitochondria in early embryos. CMXRos-loaded zygotes were photoirradiated for either 0 (P0) or 20 (P20) s to induce mitochondrial damage. Mitochondria from these zygotes were injected by cytoplasmic transfer (CT) into recipient zygotes, resulting in CT-P0 (A-D and I-L) and CT-P20 (E-H and M-P) groups, respectively. CT-derived zygotes were cultured *in vitro* for 24 h in either absence **(A–H)** or presence **(I–P)** of rapamycin. Autophagosomes in two-cell embryos were detected by immunofluorescence using a primary antibody against MAP1LC3B (green; **B,F,J,N**). Injected mitochondria were visualized based on CMXRos fluorescence (red; **A,E,I,M**). Pictures were merged to assess mitochondria-autophagosome colocalization **(C,G,K,O,D,H,L,P)**. Bars in **(A–C,E–G,I–K,M–O),** and **M–O** correspond to 10 μm, while in **(D,H,L,P),** they correspond to 2 μm.

To investigate whether autophagy was linked with the drop in the levels of photoirradiated mitochondria in blastocysts, zygotes subjected to CT were cultured in the presence of rapamycin—an autophagy agonist (Lee et al., 2011; Gilkerson et al., 2012; Dai et al., 2014). As a result, the rapamycin treatment precluded elimination of photoirradiated-derived mtDNA, resulting in similar (*P* > 0.05) levels of NZB and total mtDNA between CT-P0 and CT-P20 blastocysts (Figures 3A,B). Additionally, analysis of CT-derived embryos (at the two-cell stage) provided no evidence of increased mitochondria-autophagosome colocalization, regardless of the rapamycin treatment (Figure 4). Taken together, these results do not support a link between autophagy and elimination of photoirradiated-derived mitochondria during early embryogenesis.

## Discussion

Our present findings provide new evidence that damaged mitochondria are eliminated during early embryogenesis through an autophagy-independent mechanism.

After absorption of light, certain biocompatible photosensitizers are capable of generating reactive species (i.e., hydroxyl radicals and singlet oxygen), which may damage neighboring biomolecules such as membrane unsaturated lipids, proteins, and DNAs (Foote, 1968). Given that some photosensitizers accumulate in specific subcellular compartments, damage can be efficiently targeted to mitochondria (Gabrielli et al., 2004; Oliveira et al., 2011; Hammerer et al., 2018; Taba et al., 2018). In this respect, CMXRos has been shown, both in cultured cells and mouse oocytes, to be a potent mitochondrial photosensitizer (Minamikawa et al., 1999; Lum et al., 2002; Palermo et al., 2002; Lum and Nagley, 2003; Takeuchi et al., 2005). Hence, we have used CMXRos and photoirradiation to specifically induce mitochondrial damage in mouse zygotes. As a result, we found that either CMXRos or photoirradiation alone have no effect on development into blastocysts. Yet, photoirradiation of CMXRos-loaded zygotes for 5 s or more led to a linear decline (up to 20 s) on blastocyst rate. These results corroborate previous findings that photosensitization of oocytes leads to mitochondrial dysfunction and developmental arrest after fertilization (Palermo et al., 2002; Thouas et al., 2004, 2006; Takeuchi et al., 2005).

Given the time-depend effect of photoirradiation, we decided to photoirradiate NZB zygotes, loaded with CMXRos, for either 0 or 20 s. Next, their cytoplasm was transferred into B6 zygotes aiming to track injected mitochondria in blastocysts. Injected and recipient mitochondria were distinguished based on mtDNA origin, respectively, NZB and B6. Importantly, ∼30% of NZB mtDNA were present in zygotes, regardless of the photoirradiation treatment. However, the levels of NZB mtDNA dropped in blastocysts only when photoirradiated-zygotes were used as cytoplasmic donors. Once zygotes injected with either photoirradiated or non-photoirradiated cytoplasm developed into blastocysts with similar rates, this drop cannot be attributed to an impact of CT on embryogenesis. Moreover, mtDNA copy number remained stable between stages (zygotes vs. blastocysts) and treatments (photoirradiated vs. non-photoirradiated), indicating that photoirradiated-derived mtDNA was replaced by recipient mtDNA in blastocysts.

There is mounting evidence in support of purifying selection acting in the female germline to prevent the accumulation of deleterious mtDNA mutations (Sato et al., 2007; Fan et al., 2008; Stewart et al., 2008; Freyer et al., 2012; Sharpley et al., 2012; Li et al., 2016; Floros et al., 2018; Lieber et al., 2019; Wei et al., 2019). Among other stages of germline development, purifying selection may take place during early embryogenesis as reported by Lee et al. (2012) and Latorre-Pellicer et al. (2019). In agreement with these reports, our present data indicate that the levels of NZB mtDNA dropped in blastocysts only when it derived from photoirradiated cytoplasm. Given that photoirradiation leads to mitochondrial damage (Minamikawa et al., 1999; Lum et al., 2002; Palermo et al., 2002; Lum and Nagley, 2003; Takeuchi et al., 2005), including on mtDNA (Battogtokh et al., 2018), we argue that photoirradiated mitochondria were targeted for destruction in preimplantation embryos (Wang et al., 2012). This hypothesis is in keeping with autophagic elimination of paternal mitochondria following fertilization (Rojansky et al., 2016), suggesting that a similar mechanism might be involved with elimination of dysfunctional mitochondria inherited from the oocyte.

To address the hypothesis that photoirradiated mitochondria were destroyed by autophagy, injected embryos were cultured in the presence of rapamycin. We expected with this treatment to enhance the drop in the levels of NZB mtDNA as rapamycin is a canonical inducer of macroautophagy; by inhibiting mTORC1, rapamycin induces autophagosome formation and degradation of cellular components such as dysfunctional mitochondria (Kim et al., 2002; Narendra et al., 2008; Twig et al., 2008; Suen et al., 2010; Gilkerson et al., 2012; Dai et al., 2014). In opposite to our prediction, rapamycin prevented the drop in NZB mtDNA associated with injection of photoirradiated cytoplasm. Although difficult to explain, we propose that rapamycin mitigated mitochondrial damage induced by photoirradiation. This hypothesis is supported by a previous report showing that rapamycin upregulates DNA repair enzyme OGG1 (Habib et al., 2010). Thus, rapamycin might have countered mitochondrial damage by enhancing mtDNA repair on photoirradiated-derived mitochondria. In addition, embryos were assessed as for colocation between injected mitochondria and autophagosomes. Two-cell embryos were used as an autophagic wave takes place at this stage (Tsukamoto et al., 2008), coinciding with destruction of paternal mitochondria in mice (Rojansky et al., 2016). However, no skewed colocalization of photoirradiated mitochondria and autophagosomes was seen, even when considering the rapamycin treatment. Together, these data do not implicate autophagy in the elimination of photoirradiated-derived mitochondria.

Our current findings support the hypothesis that damaged mitochondria are destroyed during early embryogenesis, suggesting that the same mechanism might take place to counter expansion of deleterious mtDNA mutations. Such mechanism is in accordance with the “Muller’s ratchet” theory, which proposes that uniparental inheritance of mtDNA in the absence of recombination would lead to accumulation and fixation of deleterious mutations (Muller, 1964). In fact, few highly deleterious mutations in mtDNA have become fixed in the human population, lending further support to purifying selection (Rand and Kann, 1996; Elson et al., 2004; Rand, 2008; Wei et al., 2019). Considering that deleterious mtDNA mutations may impact mitochondrial function, mutations might be selected against at the organelle level (Burr et al., 2018). In support of this notion, previous reports have provided evidence that autophagy acts to eliminate dysfunctional mitochondria with deleterious mtDNA mutations (Narendra et al., 2008; Twig et al., 2008; Suen et al., 2010; Gilkerson et al., 2012; Dai et al., 2014). Although our findings do not support a link between autophagy and the lower levels of photoirradiated-derived mitochondria in blastocysts, this requires further investigation as it might be a rapamycin-independent mechanism (Yamamoto et al., 2014) or take place at a different embryonic stage (Tsukamoto et al., 2008).

## Conclusion

The preimplantation embryo is competent to mitigate the levels of damaged mitochondria. This finding is of relevance for the transmission of mitochondrial disease as a similar mechanism might take place during early embryogenesis to counter expansion of deleterious mtDNA mutations. Limitation of the study: lack of mtDNA sequencing data. Further studies are needed to show whether the lower levels of damaged mitochondria in blastocysts are linked with elimination of potentially deleterious mtDNA mutations derived from photoirradiation.

## Data Availability Statement

The datasets generated for this study are available on request to the corresponding author.

## Ethics Statement

The animal study was reviewed and approved by the Animal Care and Use Committee at Universidade de São Paulo (USP—protocol number 13.1.1832.74.8).

## Author Contributions

MRC designed the experiments and wrote the manuscript. TM, CM, MDC, and MRC carried out the experiments, data organization, and statistical analyses. FG and FM contributed new reagents and analytical tools. All authors read and approved the final manuscript.

## Conflict of Interest

The authors declare that the research was conducted in the absence of any commercial or financial relationships that could be construed as a potential conflict of interest.
